# *In vitro* production of steroidal saponin, total phenols and antioxidant activity in callus suspension culture of *Paris polyphylla* Smith: an important Himalayan medicinal plant

**DOI:** 10.3389/fpls.2023.1225612

**Published:** 2023-08-10

**Authors:** Janhvi Mishra Rawat, Shweta Pandey, Balwant Rawat, Sumit Purohit, Jigisha Anand, Arvind S. Negi, Ajay Thakur, Mohamed H. Mahmoud, Ahmed M. El-Gazzar, Gaber El-Saber Batiha

**Affiliations:** ^1^ Department of Biotechnology, Graphic Era Deemed to be University, Dehradun, Uttarakhand, India; ^2^ School of Agriculture, Graphic Era Hill University, Dehradun, Uttarakhand, India; ^3^ Department of Biotechnology, Uttarakhand Biotechnology Council, Pantnagar, Uttarakhand, India; ^4^ Genetics and Tree Propagation Division, Forest Research Institute, Dehradun, Uttarakhand, India; ^5^ Department of Biochemistry, College of Science, King Saud University, Riyadh, Saudi Arabia; ^6^ Department of Veterinary Forensic Medicine and Toxicology, Faculty of Veterinary Medicine, Alexandria University, Alexandria, Egypt; ^7^ Department of Experimental Pathology and Tumor Biology, Graduate School of Medical Sciences, Nagoya City University, Nagoya, Japan; ^8^ Department of Pharmacology and Therapeutics, Damanhour University, Damanhour, Egypt

**Keywords:** *Paris polyphylla*, dioscin, diosgenin, steroidal saponin, antioxidant activity

## Abstract

*Paris polyphylla* Smith (Melanthiaceae) family, which is native to the Himalayan region, has received a lot of attention recently due to its extensive history of usage in traditional medicine. The production of steroidal saponin from callus suspension cultures of *P. polyphylla* was observed in the current study. The current study attempted to develop a *P. polyphylla* plant callus suspension culture through optimization of cultivation technique for callus suspension, quantification of total phenolic components and estimation of the extract’s antioxidant activity. A light-yellow callus was formed within six weeks of cultivating rhizomes on Murashige and Skoog (MS) media supplemented with Thidiazuron (TDZ). Furthermore, the effect of TDZ, Methyl Jasmonate (MeJA), and Yeast Extract (YE) on callus growth, steroidal saponin (dioscin and diosgenin), total phenolic content, total flavonoids, total tannin, and total antioxidant activity was also measured. The medium containing 0.5 μM TDZ depicted the maximum callus biomass (2.98 g fresh weight). Significantly high phenolic and tannin content was observed in the MS medium containing 50 μM MeJA, whereas, no significant increase was observed in total tannin production in any treatment. Three *in vitro* assays, DPPH (2,2-diphenyl-1-picrylhydrazyl), ABTS (2,2′-azino-bis (3-ethylbenzothiazoline- 6-sulfonic acid)) and FRAP (ferric ion reducing antioxidant potential) and FC (Folin-Ciocalteu), were used to assess antioxidant potential of callus. Maximum antioxidant analysis reported in 1.0 μM TDZ (6.89 mM AAE/100 g) containing medium followed by 50 μM MeJA (6.44 mM AAE/100 g). The HPLC analysis showed a high presence of dioscin and diosgenin (5.43% and 21.09%, respectively) compared to the wild sample (2.56% and 15.05%, respectively). According to the results, callus produced on media supplemented with 50 μM MeJA have significant phenolic contents and elevated antioxidant activity; nevertheless, callus growth was greater in the presence of 0.5 μM TDZ. The findings of the current study have commercial implications since greater biomass production will result in active phytochemicals that the pharmaceutical and nutraceutical sectors are in need desperately.

## 1 Introduction

Medicinal plants are important source of secondary metabolites and have drawn much attention recently due to their immense use in the traditional healthcare system. Himalayan medicinal plants are a potential entity in this context, and most of them need effective conservation and use methods due to their current state of protection. Many plants in the Indian Himalayan Region have been micropropagated successfully using plant tissue culture techniques (Giri et al., 2011; Chen et al., 2014; Purohit et al., 2015; Raomai et al., 2015, Danu, 2016, Rawat et al., 2018). The production of secondary metabolites is now carried out using plant tissue culture methods (Guo et al., 2007; Shinde et al., 2010), pathways of plant cell biosynthesis, (Verpoorte et al., 2002), in addition to the study of metabolism, synthesis of superior plant material, choosing cell lines that produce high metabolites (DiCosmo and Misawa, 1995; Zhong et al., 1999).

Plant-based chemicals are becoming more and more popular due to their numerous remarkable medicinal properties. The production of these molecules from plant tissue cultures is crucial because intact plants only produce a small amount of these compounds. In cultivated plant cells and tissues, a variety of chemical substances are synthesized and stored, including alkaloids, saponins, carotenoids, anthocyanins, and polyphenols (Yesil-Celiktas et al., 2007; Shinde et al., 2010). Polyphenols have gained a lot of attention in recent years, owing to their important role in a variety of age-related and degenerative disorders (Brewer, 2011; Procházková et al., 2011). Polyphenols constantly offer protection by scavenging a variety of reactive oxygen species (ROS), including hypochlorous acid, hydroxyl radicals, peroxynitrite, and superoxide anion (Durazzo et al., 2019; Direito et al., 2021; Khan et al., 2021). Extracts of various Himalayan plants, which are well-known ingredients in conventional medicinal or health tonics, include phytophenols. Indian Himalaya has been known for their rich cultural, spiritual, and biodiversity values across the globe. The cure for various ailments and the use of medicinal herbs in the Himalayan region are well described in the Indian sacred text Rigveda (Ravishankar and Shukla, 2007). The 3000 years old Ayurvedic system in India has evidence of medicinal plant collection from the Himalayan region (Kumar et al., 2017). Among various medicinal plants being used for the preparation of medicines, *Paris polyphylla* from the genus Paris has its unique identity and recognition. *P. polyphylla* is a high-value medicinal plant native to the Indian subcontinent and China, where it is widely dispersed in the Yunnan-Guizhou Plateau, (Sharma et al., 2009; Payum, 2018), Manipur in India (Mao et al., 2009) and Pokhara and Kathmandu in Nepal. Apart from China, India, and Nepal, subspecies and varieties of *P. polyphylla* are well-distributed in Myanmar, Bhutan, Vietnam, Thailand, and Laos (Paul et al., 2015; Qin et al., 2018; Govaerts, 2020). All the plants belonging to the genus Paris are known for a variety of medicinal properties and can be used as medicine (Li et al., 2015) such as relieving pain, detoxification, reducing swelling, and calming the liver. Steroidal saponin is the most important bioactive compound discovered in the *P. polyphylla* rhizome, and it accounts for 80% of the total phytoconstituents (diosgenin with their glycosyl derivatives) with numerous medicinal and pharmaceutical potential (Wu et al., 2004; Guan et al., 2018).

Further, due to high demand from pharmaceutical industries, *P. polyphylla* has been overexploited from its natural resources. In addition, the slow reproduction of this species has put it in endangered status which has resulted in a sharp price rise (Rawat et al., 2023). Currently, raw materials are mostly gathered in the wild to satisfy the high demand of pharmaceutical businesses; this has had a significant negative influence on the species’ availability in its natural environment, and it has been classified as rare or uncommon in the Himalayan area (Chinmay et al., 2009). Although there are few publications on *P. polyphylla in vitro* propagation (Raomai et al., 2014; Raomai et al., 2015; Puwein and Thomas, 2022; Thakur et al., 2022), commercial *P. polyphylla* species production has not yet been documented. Therefore, plant tissue culture techniques for the optimal synthesis of worthy and advantageous metabolites with medicinal significance would be pertinent to lessen the burden on the native population. Due to the lack of callus culture research and the antioxidant properties of phenolics that have been published so far, *P. polyphylla* has been chosen for the present investigation. It should be noted that the antioxidant activities of the phenolic compounds isolated from other Himalayan medicinal plants have not been investigated, despite research on the callus culture of some of them having been published. Therefore, the current study attempted to develop a *P. polyphylla* plant callus suspension culture through (i) optimization of cultivation technique for callus suspension, (ii) quantification of total phenolic components; and (iii) estimation of the extract’s antioxidant activity.

## 2 Materials and methods

### 2.1 Plant materials and callus induction

Rhizomes of *P. polyphylla* were collected from Malyadhor, Bageshwar, Uttarakhand, India (30°08’48.8’’ N; 79°58’14.0’’ E; an altitude of 2348 m) in October 2020. Rhizomes were used as an explant to establish the callus culture. The explants were washed in flowing tap water for 20 min before being immersed in Bavistin solution (0.1%; BASF India Ltd.), containing Tween 20 (2% v/v) in distilled water. The explants were rinsed thrice with sterilized distilled water to eliminate any excess Bavistin and detergent. Moreover, explant surfaces were cleaned with mercuric chloride aqueous solution. (HgCl_2_; 0.05%, w/v; 2 min) before being repeatedly washed with sterilized distilled water in a laminar airflow (LAF) cabinet (Thermadyne, Faridabad, India). In aseptic conditions, the explants were inoculated on MS medium (Murashige and Skoog, 1962) containing TDZ (0.0 to 2.0 µM), sucrose (3%), and agar (0.8%) maintained at 5.8 pH. The inoculated agar plates were kept at 25 ± 2°C for 16 and 8 hours of light and dark cycles respectively on racks with cool fluorescent tubes (Philips 40 W; 42.0 and 60.0 mol/m2/s irradiance within and outside the culture flasks, respectively). To achieve the optimum growth, sub-culturing was carried out at 6-week intervals. Optimum growth of callus was observed on MS medium containing 0.5 µM TDZ. Further, callus was sub-cultured in ½ MS liquid medium in addition to 0.5 µM TDZ to establish callus suspension culture. All the cultures were kept in the culture room.

### 2.2 Growth curve

To generate suspension culture, 0.50 g of callus was placed into 100 mL of ½ MS liquid medium and 0.5 µM TDZ in Erlenmeyer flasks (Borosil India Ltd., Mumbai). The callus was extracted at an interval of seven days for eight weeks and filtered through Whatman No. 1 papers. The experiment was performed in triplicates. The fresh weight of the callus biomass was determined and its average was calculated. The dry weight (DW) was recorded at a temperature of 50°C in a hot air oven. The suspension of callus culture was kept in a culture chamber on a rotary shaker at 90 revolutions per min in a culture room.

### 2.3 Effect of TDZ, MeJA, and YE on callus biomass

TDZ, MeJA, and YE were used to determine their effect on the development of callus suspension. In the Erlenmeyer flasks, callus (0.5g) was placed in the100 ml of ½ MS liquid medium with increasing doses of TDZ (0.0-2.0 M), MeJA (10-200 µM), or YE (100 to 1000 mg/l). The experiment was performed in triplicates. The average callus biomass weight was evaluated after incubation of 4 weeks. The flasks without TDZ, MeJA, or YE were used as control samples.

### 2.4 Extraction and quantification of total phenol, flavonoids, tannin

The extraction of phenolic compounds from callus suspension culture was carried out to check the effect of TDZ, MeJA, or YE. The callus was thoroughly cleansed with distilled water and then allow to dry in a hot air oven at 50°C for 48 hours. After that, dried callus were mashed with a mortar and pestle and the resulting powder (100mg) was dissolved in 10 ml of 80% (v/v) ethanol for extraction at 50-60 °C for 7-8 hrs, following which the solvent was vaporized to a dry state. The residues were then dissolved in 10 ml of distilled water and used to measure the antioxidant activity, total flavonoid content, total phenolic content, and total tannin contents (Swain and Hillis, 1959; Giri et al., 2012; Raomai et al., 2015; Lepcha et al., 2019). Total phenolic content was calculated as mg Gallic Acid Equivalent (GAE)/g dry weight of sample, while total tannin content was measured as mg Tannic Acid Equivalent (TAE)/g dry weight of the sample, respectively. The estimation of total flavonoids was represented as mg quercetin equivalent/g sample.

### 2.5 Quantification of steroidal saponin

The plant samples were analyzed for dioscin and diosgenin (steroidal saponin) content following the method of Raomai et al. (2015). For diosgenin, the plant sample was prepared by refluxing the ground plant material with 50 ml H_2_SO_4_ for 4 hours and further extracted with 50 ml Ethyl acetate in a separating funnel thrice. The organic layer was collected and passed over anhydrous NaSO_4_ and evaporated. The residue was dissolved in ethanol and filtered with 0.45-micron filter paper. This filtrate was further used for the quantification of diosgenin.

For dioscin, ground sample (5 gm) was mixed with ethanol-water mixture (7:3 v/v), and incubated at 50°C for 15 min. The mixture was then sonicated in the ultra-sonicate bath for 15 min, centrifuged and the supernatant was collected and filtered with 0.45 micron filter paper. This filtrate was further used for the quantification of dioscin.

Ethanolic extract was employed in an HPLC analytical system (Merck Hitachi, Japan). The samples were separated in an isocratic manner using an isocratic mobile phase comprising acetonitrile: ethanol in a ratio of 80:20 v/v. The flow rate was kept at 1 ml/min in the C-18 column (Purosphere, Merck). The volume of the sample used was 20 µl. The retention duration of comparable external standards was used to identify phenolic chemicals. The compounds were identified with the help of retention time of reference standard. Diosgenin or dioscin were quantified with help of calibration curve of peak areas of external standards recorded at 196 nm. The standard curve of peak area for each compound was prepared at five different concentrations ranging from 05 to 20 µg/ml and linear regression curve between peak area and concentration was used for quantification (**Supplementary File S1**). The results were expressed in mg/100 g or µg/g.

### 2.6 Antioxidant activity

Three *invitro* methods were followed for the assessment of the antioxidant activity of the samples. viz ABTS assay, DPPH assay and FRAP assay.

#### 2.6.1 ABTS assay

The total antioxidant activity was determined using the ABTS (2,2’-azino-bis (3-ethylbenzothiazoline-6-sulfonic acid) free radical scavenging assay with slight modifications (Cai et al., 2004). To create ABTS cation (ABTS•+), 7.0 M ABTS and 2.45 M Potassium persulfate were combined and left in the darkness for 16 hrs at 22°C. The ABTS•+ was diluted in 80% (v/v) ethanol till the absorbance of 0.70 (0.05) at 734 nm in a UV-Vis Spectrophotometer (Hitachi, Japan) was recorded. For the assay, diluted ABTS•+ solution and methanolic extract were thoroughly combined. The reaction mixture was left undisturbed for a suitable amount of time (at 22 ± 1°C for 6 min in the darkness), before recording the absorbance at a wavelength of 734 nm. The calibration curve was plotted using varying concentrations of standard Ascorbic acid. The measurements were represented in mM Ascorbic acid Equivalent/100 g dry weight of the sample.

#### 2.6.2 DPPH assay

The standard method of DPPH (2,2-diphenyl-1-picrylhydrazyl) free radical scavenging assessment with slight modification was used for the determination of antioxidant activity (Brand-Williams et al., 1995). Solution of 100 mM DPPH was prepared in 80% ethanol (w/v), and combined with sample extract, kept in the darkness at 22°C for 20 min. The absorbance was measured in a UV-Vis spectrophotometer at the wavelength of 520 nm and the data were represented as mM Ascorbic acid Equivalent/100 g dry weight of the sample.

#### 2.6.3 FRAP assay

The FRAP (Ferric reducing anti-oxidant power) experiment with some modifications was performed for the evaluation of antioxidant activity (Benzie and Strain, 1996). One vol. of 2,4,6-tri-2-pyridyl-1,3,5-triazine (TPTZ) mixed with 40 mM HCl, 10 vol. of 300 mM acetate buffer, (16 ml Glacial Acetic Acid/L and 3.1 gm Sodium Acetate), and 01 vol. of 20mM FeCl_3_ were combined to create the FRAP reagent.

The mixture of methanolic extract and FRAP reagent was preheated to 37°C and held for 8 min. The absorbance at the wavelength of 593 nm was measured in a UV-Vis Spectrophotometer. A standard calibration curve was prepared using varying concentrations of standard ascorbic acid, and the data were represented as mM Ascorbic Acid Equivalent/100 g of fresh weight.

### 2.7 Design of experiment and data analysis

**Figure 1:**
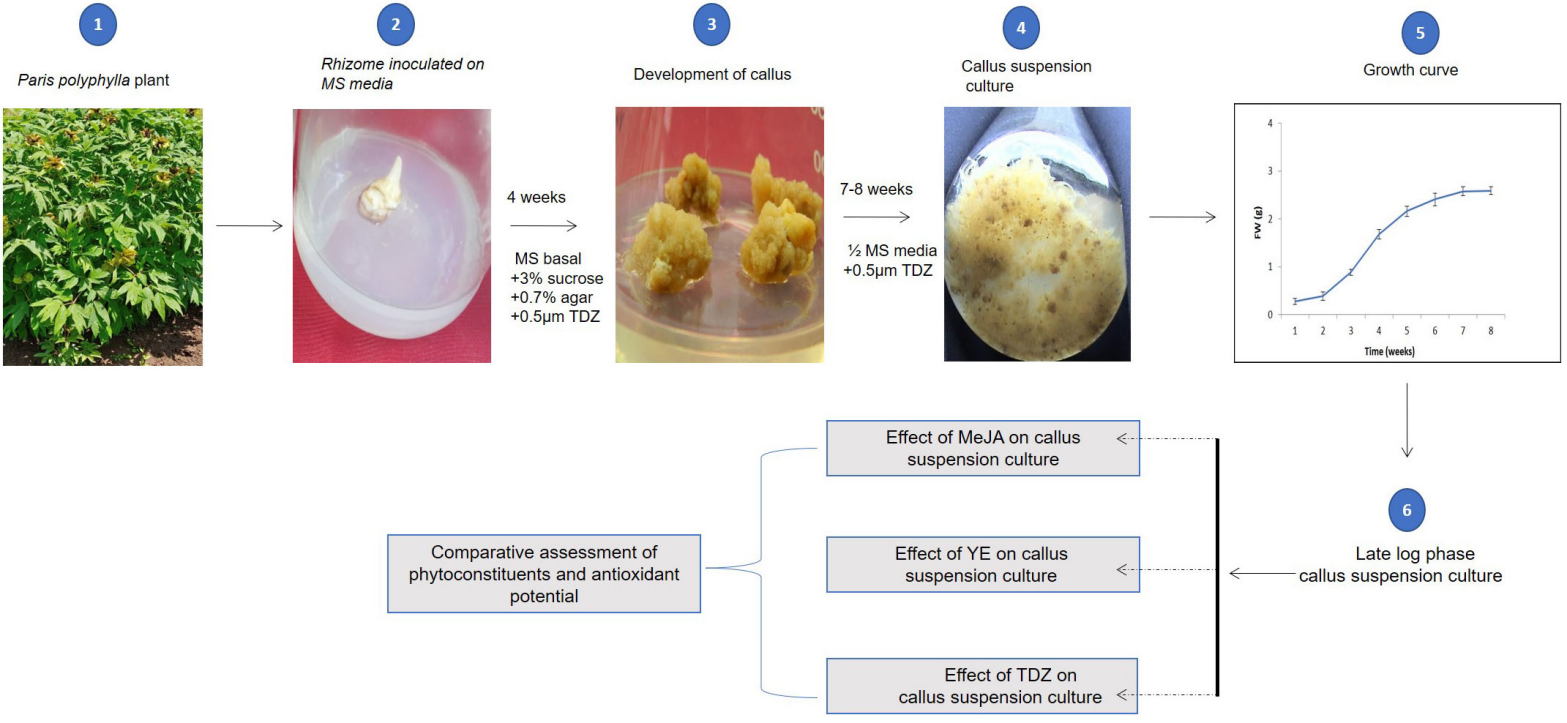
Graphical representation of complete methodology used to establish callus culture of *P. polyphylla*, to analyse effect of MeJA, YE and TDZ and evaluation of phytoconstituents and antioxidant potentials.

Complete experimental design is presented in **Figure 1**. The experiments were conducted twice in a randomized block design, with triplicates for each treatment. Mean values from various treatments were assessed using SPSS version 7.5. The difference in the mean values was determined at significance (at p 0.05) using Duncan’s Multiple Range Test (DMRT).

## 3 Results and discussion

### 3.1 Callus culture

Within 6 weeks of inoculation, rhizomes transplanted to MS medium containing 0.5 µM TDZ displayed brittle and light-yellow colored calli. These rhizomes derived callus were further transferred in liquid MS media supplemented with 0.5 µM TDZ. The outcomes show that *P. polyphylla* rhizome-derived callus grows at its best on MS medium containing 0.5 µM TDZ. Similar outcomes have been seen with callus induction in seeds of several medicinal plants that needed plant growth regulators (PGRs; Hong et al., 2008; Giri et al., 2012). These rhizomes derived callus were subsequently used to plot a growth curve and carry out additional experiments.

### 3.2 Growth curve

**Figure 2:**
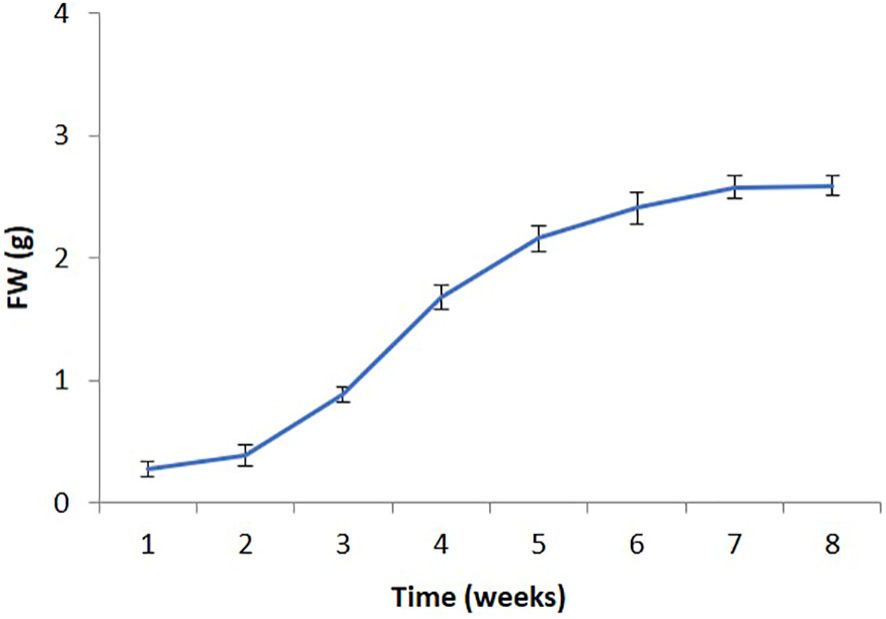
Growth curve of *P. polyphylla* (callus suspension culture) on MS liquid medium supplemented with 0.5 µM TDZ. Values are mean ± SE of three replicates.

Callus development on the MS medium containing 0.5 µM TDZ for 8 weeks of culture exhibited a 15-fold increase in biomass (fresh weight). The study revealed 7 days of a lag phase, a log phase extending from 7 to 30 days and lastly, a stationary phase was present (**Figure 2**). The late log phase callus (after 32 days), was employed for further examinations.

### 3.3 Effect of TDZ, MeJA, and YE on callus biomass

The influence of varying concentrations of TDZ (0.0-2.0 µM), MeJA (10-200 µM), or YE (100 to 1000 mg/l) on callus biomass were examined (**Table 1**). Higher callus biomass resulted from the addition of TDZ. The best TDZ concentration for biomass production was 0.5 µM, however, higher TDZ concentrations (2.0 µM) harm biomass output.

**Table 1:** Effect of different concentration of TDZ, MeJA and YE concentrations on callus growth, total phenol, total flavonoids, total tannin and antioxidant potential of callus suspension culture of *P. polyphylla*.

Treatments	Growth (g FW)	Total phenol (GAE/ g DW)	Total flavonoids (QE/g DW)	Total tannin (TAE/ g DW)	Antioxidant analysis (mM AAE/100 g)		
TDZ (µM)					DPPH	ABTS	FRAP
0.0	1.02± 0.04^d^	18.66± 1.64^e^	28.43± 1.94^b^	43.66± 3.22^c^	1.09± 0.06^e^	1.71± 0.09^e^	1.46± 0.02^c^
0.1	1.82± 0.06^b^	24.33± 2.22^d^	29.88± 1.55^b^	44.23± 3.34^c^	1.43± 0.08^d^	4.26± 0.29^c^	1.66± 0.83^b^
0.5	2.98± 0.08^a^	37.66± 2.95^b^	33.67± 2.06^a^	48.45± 4.88^b^	1.61± 0.08^c^	4.56± 0.33^c^	1.68± 0.66^b^
1.0	2.64± 0.08^a^	38.23± 3.06^b^	34.66± 2.12^a^	48.67± 3.56^b^	1.82± 0.09^c^	6.89± 1.02^a^	1.46± 0.02^c^
2.0	2.33± 0.09^ab^	37.43± 3.17^b^	34.54± 3.04^a^	46.86± 6.44^b^	1.67± 0.07^c^	4.16± 0.47^c^	1.79± 1.03^a^
MeJA (µM)							
10	1.22± 0.09^c^	32.33± 2.25^c^	30.43± 3.07^b^	45.33± 4.86^c^	1.23± 0.43^d^	3.36± 1.06^b^	1.32± 0.03^c^
20	1.92± 0.04^b^	48.34± 2.66^a^	34.58± 3.13^a^	55.45± 4.17^a^	1.66± 1.07^c^	4.54± 1.29^c^	1.65± 0.04^b^
50	2.88± 1.02^a^	49.66± 4.43^a^	34.67± 3.22^a^	57.66± 4.22^a^	2.21± 1.09^a^	6.44± 1.09^a^	2.88± 0.08^a^
100	1.34± 0.09^c^	39.78± 3.23^b^	32.66± 2.84^a^	48.89± 3.55^b^	2.02± 0.97^b^	4.86± 1.89^c^	1.89± 0.23^b^
200	1.66± 0.05^bc^	31.44± 2.76^c^	32.44± 2.92^a^	47.34± 3.33^b^	2.09± 0.88^b^	4.16± 1.09^d^	1.46± 0.65^c^
YE (mg/l)							
100	1.44± 0.05^c^	21.55± 1.29^d^	31.23± 3.11^ab^	42.23± 4.19^c^	1.09± 0.07^e^	3.33± 0.89^b^	1.22± 0.03^c^
250	2.82± 1.09^a^	29.36± 2.24^c^	32.67± 3.32^a^	46.78± 4.19^b^	1.33± 0.07^d^	5.56± 0.89^b^	2.65± 1.03^a^
500	1.38± 0.13^c^	26.45± 2.04^d^	31.54± 3.19^ab^	46.45± 4.19^b^	1.22± 0.07^d^	4.56± 0.89^c^	1.35± 0.65^c^
750	1.55± 0.16^bc^	22.67± 1.79^d^	20.67± 1.32^c^	42.56± 3.88^c^	1.02± 0.06^e^	3.88± 0.09^d^	1.06± 0.44^d^
1000	1.32± 0.19^c^	22.45± 1.86^d^	20.54± 2.09^c^	43.84± 3.11^b^	1.19± 0.06^e^	1.21± 0.09^e^	1.22± 0.24^c^

Data are mean of three replicates each with ten explants, ± Standard Error, values with different letters are significantly different (p < 0.05).

An analysis of MeJA’s impact on callus biomass was also conducted. MeJA was observed to harm biomass production at higher concentrations (200 µM), and it was not found to be effective in increasing biomass. Elicitors like MeJA have been demonstrated to regulate the mitotic cycle in plant cells, restricting cell division and biomass increase by preventing cell division in the G1 phase before entering the S phase as signalling molecules in the plant defence system. As a result, under high MeJA concentrations, biomass production declines or ceases entirely (Kang et al., 2004; Ho et al., 2018; Mendoza et al., 2018). YE displayed a similar pattern; at a dosage of 250 mg/l, it promoted callus growth and boosted biomass production, however at 1000 mg/l, the biomass was reduced to 1.32 g FW at 2.5 mg/l (**Table 1**).

### 3.4 Effect of TDZ, MeJA, and YE on total phenol, flavonoids, and tannin

All three components i.e., total phenol, flavonoids, and tannin showed increased amounts with increasing TDZ concentration (**Table 1**). While the highest quantity of total phenol (49.66 ± 4.43 GAE/g DW), flavonoids (34.67 ± 3.22 QE/g DW), and tannin (57.66 ± 4.22 TAE/g DW) all had been recorded at 50 µM MeJA, the maximum amount of biomass production (2.98 ± 0.08), flavonoids (34.67 ± 3.22 QE/g DW), and tannin were all observed at 0.5 µM TDZ.

According to studies by Jalalpour et al. (2014) and Ayoola-Oresanya et al. (2021), MeJA was responsible for increasing phenolic and flavonoid accumulation in *Taxus baccata* cell culture. Earlier, MeJA greatly increased the amount of alkaloid in *O. liukiuensis* hairy roots produced when they were elicited in a liquid medium (Asano et al., 2004). However, a further rise in MeJA concentration (200 µM) harms the production of biomass, phenol, flavonoids, and tannin (**Table 1**). It should be noted that the use of PGRs and elicitors to examine callus biomass production and the build-up of secondary metabolites in a variety of species led to equivalent results, (Sudha and Ravishankar, 2003; Liu et al., 2007; Yesil-Celiktas et al., 2007; Giri et al., 2012).

Nevertheless, elicitation by yeast extract had a suppressive effect on the synthesis of total phenol, flavonoids, and tannin. Several workers have also noted comparable outcomes (Asano et al., 2004; Xu et al., 2016; Pisitpaibool et al., 2021). **Table 1** compares the effectiveness of each treatment with the control.

### 3.5 Antioxidant activity

All the phenolic substances discovered in this investigation have the potential to act as antioxidants. The biological effects of phenolic compounds, alkaloids, flavonoids, and tannins found in plants include anti-cancerous, anti-inflammatory, and antioxidant properties, among others (Miller, 1996; Nithya et al., 2016; Muniyandi et al., 2019). Three separate *in vitro* experiments used to determine the antioxidant activity of all phenolics revealed diverse results (**Table 1**). With treatment with 50 µM MeJA, the highest level of antioxidant activity as measured by DPPH, ABTS, and FRAP was observed. The maximal antioxidant activity was estimated to be 6.89 mM Ascorbic acid Equivalent/100 g dry weight of the sample as depicted by ABTS assay, compared to 2.21 mM Ascorbic Acid Equivalent/100 g of dry weight for the DPPH assay. For the FRAP experiment, 50 µM MeJA was reported to demonstrate the highest antioxidant activity (2.88 mM Ascorbic Acid Equivalent/100 g of dry weight). All three assays followed depicted the highest antioxidant activity at 50 µM MeJA, however, these assays resulted in different ranges of antioxidant potential for each experiment. The variations observed in antioxidant activity as observed by multiple tests are most likely connected to the respective modes of action of three assays. The reduction of a ferric analog is the foundation of the FRAP assay. In the presence of any antioxidant, the tripyridyl-triazine Fe (TPTZ)+++ Fe+++ complex is transformed into a vivid blue color Fe (TPTZ)++ at low pH (Wong et al., 2006). Differences in antioxidant activity have been found by other researchers as a result of the different techniques used (Surveswaran et al., 2007; Rawat et al., 2011; Giri et al., 2012).

In the control, reduced antioxidant activity was determined by all three of the assays used in the study. Minimum antioxidant activity was recorded with DPPH assay (1.09 mM Ascorbic Acid Equivalent/100 g of dry weight) and maximum was recorded for ABTS assay (6.89 mM Ascorbic Acid Equivalent/100 g of dry weight). Similar results have been reported in recent studies (Jalalpour et al., 2014; Makowczyńska et al., 2015; Ayoola-Oresanya et al., 2021).

### 3.6 Comparative evaluation of phytoconstituents and antioxidant potentials

The antioxidant potential and total phenolic contents of the *in vitro* generated callus *(*best cultures of TDZ, MeJA, and YE) and control were considerably different. Total phenol concentration in *invitro* callus suspension culture was more than twice higher than in control (**Table 1**). In addition, compared to the control, the flavonoids and tannin levels dramatically increased. TDZ and MeJA grown callus were shown to have significantly (p 0.05) higher antioxidant activity in comparison with control (reported as mM Ascorbic Acid Equivalent/100 g of dry weight). However, using the DPPH assay, no appreciable difference in antioxidant activity was found. Many studies have shown how different PGRs and elicitors can be used to produce or augment secondary metabolites and other advantageous bioactive compounds (Giri et al., 2012; Jalalpour et al., 2014; Makowczyńska et al., 2015; Ayoola-Oresanya et al., 2021). The elicitor MeJA (50 µM concentration) was found to be quite successful at increasing the total phenol, flavonoids, and tannin content in *P. polyphylla*, whereas TDZ was useful in improving callus biomass.

### 3.7 Comparison of steroidal saponin content

The production of steroidal saponin (dioscin and diosgenin) from wild rhizomes and *in vitro* callus (the best cultures of TDZ, MeJA, and YE) were compared. The concentration of dioscin in callus cultures treated with 1.0 µM TDZ and 50 µM MeJA was reported to be more than two times greater.

**Figure 3:**
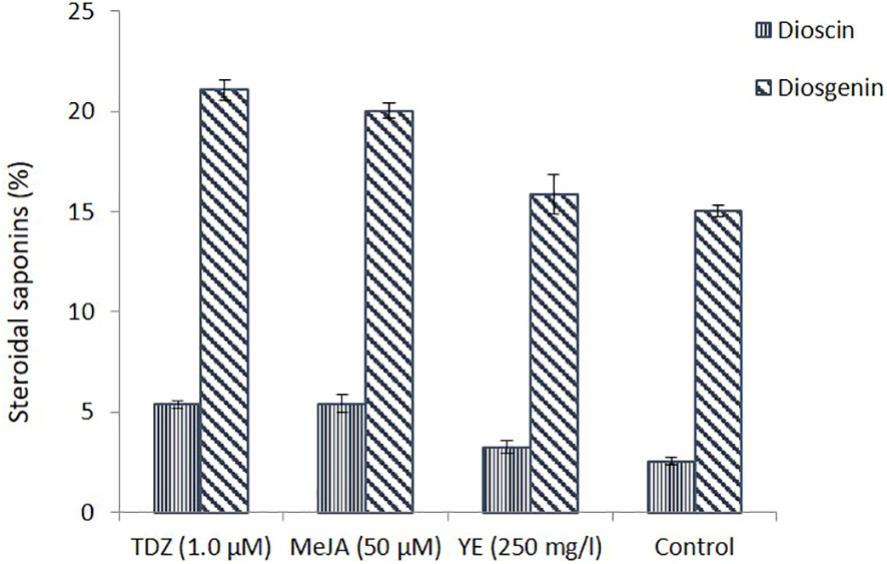
Steroidal saponins (dioscin and diosgenin) estimation in callus suspension culture (under different treatments) and in control.

**Figure 4:**
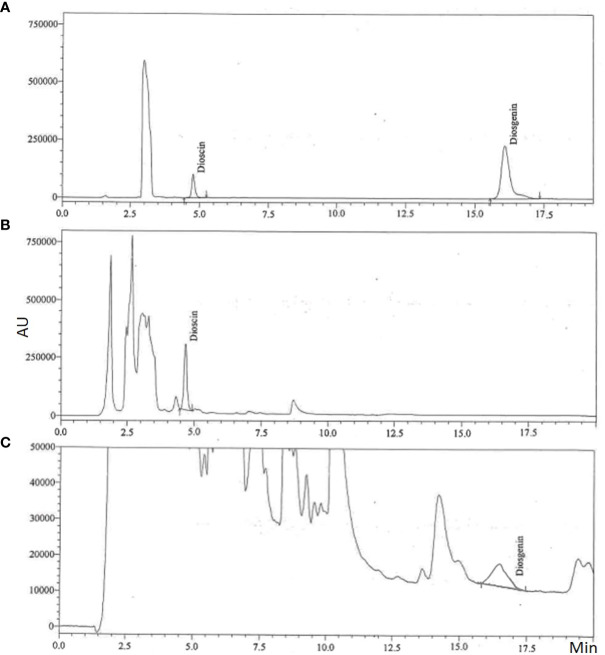
Representative chromatograms of steroidal saponin (dioscin and diosgenin); **(A)** Standard, **(B)** Dioscin in *in vitro* sample and **(C)** Diosgenin in *in vitro* sample.

MeJA is efficient in increasing the production of secondary metabolites in cell cultures and is essential for signal transduction processes that control plant defence responses (Zhao et al., 2005; Singh and Dwivedi, 2018). Although YE indicated an increase in dioscin production, it was not substantial. Similar findings were observed for the generation of diosgenin content, which was considerably higher than that of control (**Figure 3**). Chromatogram of steroidal saponin diosgenin and dioscin is also presented in **Figure 4**. According to the culture method, Wang et al. (2022) recently observed that the MeJA-treated Chinese chives had substantial variations in primary and secondary metabolites. Similar outcomes were also reported by other researchers, who claimed that using MeJA as an elicitor in the culture increased the synthesis of the secondary metabolites (Rodríguez-Sánchez et al., 2020; Deveci et al., 2022; Wang et al., 2022).

## 4 Conclusion and future perspectives

Heavy extraction of medicinal plants from the wild, loss of habitat through destruction and excessive grazing pressure at high altitude have limited the regeneration of these plants and, therefore survival of these medicinal plant is under threat. It is therefore, imperative to look for alternative sources of such types of compounds. The development of callus suspension cultures has led to the commercial production of several pharmaceutically important compounds.

There are many reports on *in vitro* propagation and secondary metabolite production of *P. polyphylla* (Raomai et al., 2014; Raomai et al., 2015; Puwein and Thomas, 2022; Thakur et al., 2022). The effect of elicitors to enhance the steroidal saponins in mini-rhizome cultures of *P. polyphylla* has also been reported by Raomai et al. (2015), however, callus suspension culture and their phytochemical analysis has reported in the current study. The current investigation demonstrated the variation in steroidal saponins between control and *in vitro* cultures. The *in vitro* process discussed here can also be used to produce secondary metabolites in a constrained amount of time and space. The current findings can be used for additional research to comprehend alternate strategies for producing secondary metabolites.

Further, to isolate, purify, and characterise the phytochemical components responsible for the pharmacological effects, more research should be done. It is possible to find novel medicines by using the phytochemical components of *P. polyphylla* and their derivatives.

Additionally, the development of an alternative source of active compounds from the establishment of callus suspension cultures of significant medicinal plants can lessen the pressure on the natural plant population while also providing the state with additional economic benefits through commercial applications. Moreover, establishment of callus suspension culture of important medicinal plant will not only reduce the pressure on the natural plant population but can bring more economic benefits to the state by way of commercial applications. Despite the species’ significant contribution to health foods previously, the current finding of this study has enormous commercial ramifications as it may supplement the expanding need of the pharmaceutical sectors. Furthermore, attention should be paid to the information generation on different aspects in achieving conservation and sustainable utilization of this important medicinal plant. This study will ensure the identification of the best propagation protocol system in both *in vitro* and *ex vitro* conditions to ensure the mass production of both quality plants as well as secondary metabolites.

## Data availability statement

The raw data supporting the conclusions of this article will be made available by the authors, without undue reservation.

## Author contributions

BR, ShP, JR, and SuP: Experiment design and data collection. JA, AN, AT: Critical revision from draft to version to be published. BR, MM, AE, GE-B: Reviewed and approved the complete draft of the manuscript. All authors contributed to the article and approved the submitted version.
