# Supplementary material for: In vitro production of steroidal saponin, total phenols and antioxidant activity in callus suspension culture of Paris polyphylla Smith: an important Himalayan medicinal plant
Source: Front Plant Sci. 2023 Aug 10;14:1225612. doi: 10.3389/fpls.2023.1225612 (PMC10470836; doi:10.3389/fpls.2023.1225612)
Supplement: Supplementary file 1 [file DataSheet_1.docx]

**Supplementary file S1:** Standard calibration curve of Dioscin and Diosgenin


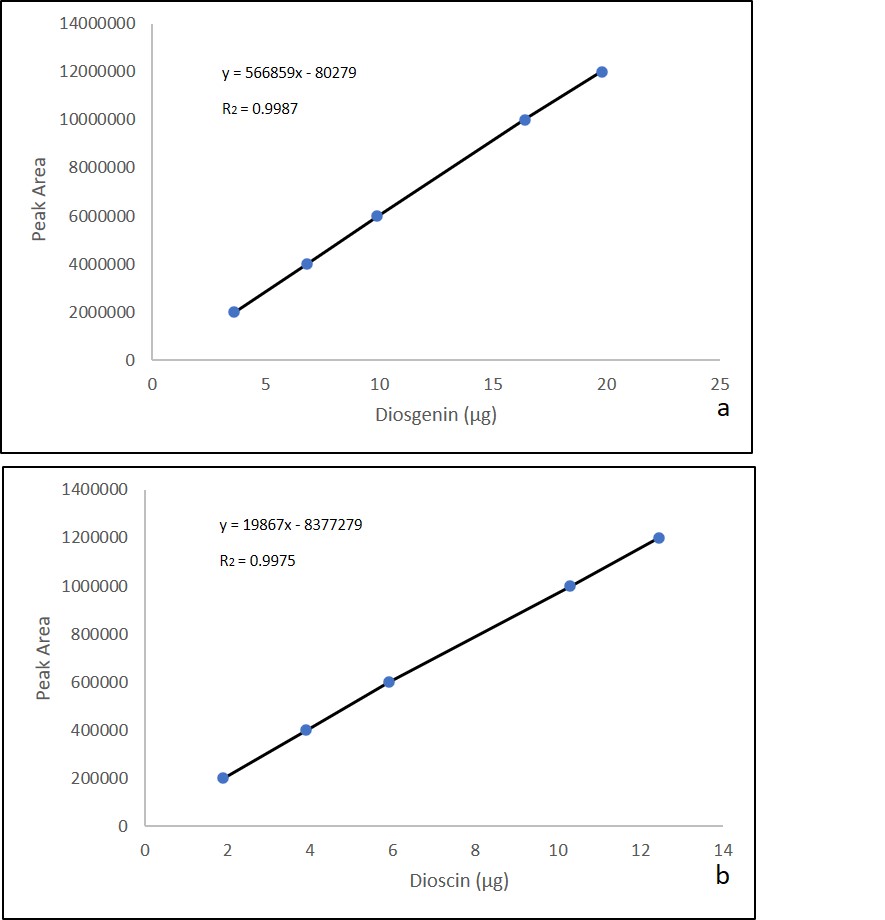


The compounds were identified with the help of retention time of reference standard. Diosgenin or dioscin were quantified with help of calibration curve of peak areas of external standards recorded at 196 nm. The standard curve of peak area for each compound was prepared at five different concentrations ranging from 05 to 20 µg/ml and linear regression curve between peak area and concentration was used for quantification. The results were expressed in mg/100 g or µg/g.

Calculation of active content in the solution was done as follows:

[C X D]/ [W X 10,000]

Where, C is concentration of active constituent

D is dilution factor of sample

W is weight of sample in g
